# A self-rating scale for patient-perceived side effects of inhaled corticosteroids

**DOI:** 10.1186/1465-9921-7-131

**Published:** 2006-10-24

**Authors:** Juliet M Foster, Eric van Sonderen, Amanda J Lee, Robbert Sanderman, Antoon Dijkstra, Dirkje S Postma, Thys van der Molen

**Affiliations:** 1 Department of General Practice and Primary Care, University of Aberdeen, UK; 2 Department of General Practice, University Medical Center Groningen, University of Groningen, The Netherlands; 3 Northern Center for Healthcare Research, University Medical Center Groningen, University of Groningen, The Netherlands; 4 Department of Pulmonary Diseases, University Medical Center Groningen, University of Groningen, The Netherlands

## Abstract

**Background:**

Patient-reported side effect questionnaires offer a simple method for the systematic measurement of drug-related side effects. In order to measure patients' inhaled corticosteroids (ICS) related side effect perceptions the 14-day retrospective Inhaled Corticosteroid Questionnaire (ICQ) was developed. In this research we aim to assess the construct validity and reliability of the ICQ and test its responsiveness to dose changes in adult asthma patients.

**Methods:**

In a cross-sectional study, current inhaler users with asthma completed the ICQ (27 with non ICS inhaler; 61 BDP equivalent daily ICS low dose ≤400 μg; 62 mid dose 401–800 μg; and 105 with high dose >800 μg). We generated 3 construct validity hypotheses: 1) a hierarchical dose-response pattern for scoring of the individual items on the ICQ, and statistically significant differences in the scores of each of the 15 ICQ domains by ICS dose group 2) an association between ICS dose and ICQ scoring after adjusting for appropriate confounders in multiple regression; 3) greater convergence between local side effect domains than between systemic and local domains of the scale. Test-retest reliability was assessed on a randomly selected subgroup of patients (n = 73) who also completed the ICQ a second time after 7 days. In a separate longitudinal study, 61 patients with asthma completed the ICQ at baseline and after changing their daily ICS dose, at 2- and 6- months, in order to test the ICQ's responsiveness.

**Results:**

All three construct validity hypotheses were well supported: 1) a statistically significant difference existed in scores for 14 domains, the high ICS dose group scoring highest; 2) ICS dose independently predicted ICQ scoring after adjusting for confounders; 3) greater convergence existed between local ICQ domains than between local and systemic domains. The ICQ had good reproducibility: test-retest intraclass correlation coefficients were ≥0.69 for all but the 'Facial Oedema' domain. In the longitudinal study, ICQ scores for 'Voice Problems' changed significantly at 2- and 6-months from baseline and other ICQ domains displayed trends in scoring change accordant with dose modulation at 6-months.

**Conclusion:**

The ICQ has good dose-related discriminative properties, is valid, reliable, and shows potential responsiveness to ICS dose change.

## Background

Drug side effects are of considerable concern to patients [1,2]. Inhaled corticosteroids (ICS), which are effective and widely recommended for controlling airway inflammation in asthma, are also known to cause many local and systemic side effects [3-6]. A crucial chasm may exist between doctors and patients with respect to their approach to drug side effects. On the one hand, doctors may avoid discussing patients' aversions to prescribed medicines [7], and on the other hand patients independently modify their treatment regimes due to concerns about potential or perceived side effects without informing their doctor [1,8]. It is perhaps unsurprising then, that drug side effects are associated with non-compliance to prescribed medication regimes in asthma [9-12], and to poor asthma outcomes [13,14].

Patient-centered self-report questionnaires need to be properly developed, validated and widely used to permit the measurement of patient-perceived side effects in the context of real-life practice, clinical trials and other research. These instruments may also provide a systematic method for the exploration of associations between side effect perceptions and medication taking behavior or other important health-related outcomes. Few drug side effect questionnaires exist, probably due to the lack of a clearly defined methodology for the development of such complex instruments. Those that are frequently cited are predominantly used for the measurement of psychoactive drug side effects (Udvalg for Klinische Undersøgelser (UKU)[15,16], Liverpool University Neuroleptic Side Effect Rating Scale [17]) and many still require further validation work.

We developed the Inhaled Corticosteroid Questionnaire (ICQ) using a combination of well-established methods from the fields of health status and psychological assessment scale development [18],.{Ghiselli, 1981 420/id}, [20]. In our previous work we used qualitative methods to generate the 57 side effect items included in the 15 domains of the ICQ (table 2), and in subsequent cross-sectional testing we demonstrated the face validity of the scale in 395 inhaler users [3]. The ICQ covers a range of patient-perceived side effects including voice, throat, skin and mood problems. The questionnaire then underwent linguistic validation for translation into 19 languages (carried out by Mapi France), with international harmonization resulting in some minor changes to the wording of the original English version (current questionnaire on our website [21]). The aims of the two studies presented in this current paper are four-fold. In the first study we empirically construct the domains of the ICQ, and test the construct validity and reliability of the full ICQ and respective domains. In the second study we examine the responsiveness of the questionnaire to change in ICS dose.

**Table 2:** The 15 domains and 57 items of the ICQ scale.

**Voice Problems – 15 items:**	**Mood Problems – 3 items:**
• hoarseness of the voice	• feeling 'grumpy'
• a 'rough' voice	• mood swings
• a noticeable change to your voice	• feeling 'easily irritated'
• your voice feeling similar to how your voice feels when recovering from the flu	
• your voice feeling like it had 'gone to the back of your throat'	**Taste Disruption – 3 items:**
• not being able to sing	• a change in your ability to taste
• loss of speech volume so that you couldn't talk as loudly as normal	• a loss of ability to taste
• a feeling of exhaustion when talking	• a loss of appetite
• a painful throat when talking	
• a feeling that other people couldn't understand your speech because you speak too softly or not clearly enough	**Perspiration – 2 items:**
• a breaking voice	• sweating
• a 'rough' throat	• sweating during the night
• a sore throat	
• an unpleasant feeling in your throat	**Oropharyngeal Itching – 2 items:**
• a dry throat	• an itchy feeling on the roof of your mouth
	• an itchy feeling in the back of your throat
**Oropharynx Problems – 9 items:**	
• coughing	**Thirst – 2 items:**
• coughing up phlegm	• feeling thirsty
• coughing up thick mucus	• wanting to drink liquid (because of a dry mouth)
• thick mucus coming up	
• thick mucus sticking at the back of your throat	**Tiredness – 2 items:**
• a need to clear your throat	• difficulty sleeping
• mucus in your throat	• feeling tired
• a 'clump' in your throat	
• a feeling that 'a layer of mucus stays on the back' of your throat	**Oral Candidiasis – 1 item:**
	• oral thrush (fungal infection: sore throat covered with pustules, and difficulty swallowing)
**Unpleasant Taste – 7 items:**	
• a terrible taste in your mouth	**Facial Oedema – 1 item:**
• a 'taste' on the teeth	• a swollen face or fluid around the face
• a 'bad taste' or unfresh feeling in your mouth	
• bad breath	**Vision Deterioration – 1 item:**
• wanting to rinse your mouth	• some kind of deterioration of your vision
• wanting to brush your teeth	
• wanting to chew gum	**Dental Deterioration – 1 item:**
	• any form of dental decline (tooth decay, tooth staining etc.)
**Skin, Hair and Nails – 7 items:**	
• dry skin	**Eye Dryness – 1 item:**
• dry skin on the face	• dry eyes
• bruising easily	
• bruises that are painful for a long period	
• thinner skin or less flexibility in your skin	
• brittle nails, or your nails breaking easily	
• hair loss	

Stem question: How much have you been affected by the following side-effects related to the use of your inhaler/s during the last 14 days?

## Methods

### Study 1 – Domain construction, construct validity and reliability of the ICQ

Ethical approval was not required for this study which required only questionnaire completion.

#### Patients

Contactable patients from 3 existing North Netherlands asthma cohorts, with a physician diagnosis of asthma and hyperresponsive to histamine (30 seconds method; PC_20 _< 32 mg/ml) were invited to participate in the study. Included patients were consenting current inhaler users, aged 16 to 74 years, with <20 pack years. Excluded patients used >1 course of oral steroids in the past 3 months and/or any oral steroid use 4 weeks prior to study, a steroid injection in the previous 6 months or any change in their ICS regimen in the previous 14 days.

Before analysis participants were divided into four groups based on their daily ICS dosage: non ICS inhaler, low dose ICS (≤400 μg), mid dose ICS (401–800 μg), high dose ICS (>800 μg)[22]. All stated ICS doses are BDP equivalent where 1 μg of beclomethasone dipropionate/budesonide is equivalent to 0.5 μg fluticasone propionate irrespective of delivery device used [23,24].

#### Study questionnaire

Patients each completed an identical self-report questionnaire at home, which elicited data on:

1. Medication use: daily use of inhaled asthma medication (ICS, Short-acting β_2_-agonist (SABA), long-acting β_2_-agonist (LABA)); current use of other steroid medication (oral tablet, nasal, ocular, dermal, eye, ear drops or creams); prednisone courses in previous three years; steroid injections in previous 6-months; current use of additional prescribed medications.

2. Inhaler behavior: spacer use; post-inhalation mouth rinsing.

3. Date of starting ICS.

4. Perceived ICS side effect: Inhaled Corticosteroid Questionnaire (ICQ).

5. Asthma severity and history: 6-item Asthma Control Questionnaire (ACQ) scored 0–6 (Forced expiratory volume in one second, question omitted)[25]; number of emergency GP appointments for asthma in the last year; age asthma diagnosed.

6. Personality: Neuroticism scale of the Eysenck Personality Questionnaire Revised Short Scale (EPQ-RSS)[26] scored 0–12; Negative affect scale of the Positive and Negative Affect Schedule (PANAS)[27] scored 10–50.

7. Patient demographics: age; gender; smoking status; educational level.

All returned questionnaires were checked for missing responses or inconsistencies, and queries were resolved with the patient by telephone.

#### ICQ domain construction procedure and analysis

We chose initial factors with an eigenvalue of greater than 1 in principle component analysis which were above the inflection point of the Cattell Scree plot [28]. Subsequently inter-item correlations of ≥0.50 were identified to cluster remaining items.

#### ICQ endorsement and scaling procedure and analysis

The percentage of patients endorsing (scoring ≥1 on the scale) each ICQ item and using each response option was calculated.

#### Construct validity procedure

We used cross-sectional construct validity to test the validity of the ICQ. The construct validity method is applied when developing a test of a construct ('construct' refers to the measured characteristic – in this case perceived side effect) for which no other measure exists. Construct validity can be undertaken by generating and empirically testing a number of hypotheses, based on what is already known about the construct [19]. We developed the following hypotheses based on their supporting rationales:

##### Rationale 1

The literature suggests that a dose-response can be expected for side effects related to the use of ICS, with side effects most likely to occur in higher doses [6].

##### Hypothesis 1

There is a hierarchical dose-response pattern for prevalence of the individual items on the ICQ and statistically significant hierarchical differences in scores for each of the 15 domains – with the high ICS dose group scoring highest on the ICQ.

##### Rationale 2

A scale measuring current side effects of ICS should be predicted by the current dose of ICS used. However, disease severity might also be associated with greater ICQ scoring. Thus, in order to demonstrate that ICQ scores are predicted by ICS dose, irrespective of severity, the ICQ scores of patients with well-controlled (mild) disease must still be independently associated with ICS dose.

##### Hypothesis 2

ICS dose independently predicts ICQ scoring after adjusting for confounders, an association which remains in patients with homogenous disease.

##### Rationale 3

Local side effects are caused by the action of steroid in the oral-pharyngeal space, whereas systemic side effects occur due to steroid absorbed into the systemic circulation. These disparate mechanisms suggest stronger associations among items in domains with a potentially homogeneous route of action than those with a potentially heterogeneous route of action.

##### Hypothesis 3

There is greater convergence (that is, stronger association) among items in ICQ domains caused by a potentially homogenous route of action (e.g. the local side effect domains 'Voice' and 'Oropharynx problems') than among items in domains caused by heterogeneous routes (e.g. 'Oropharynx Problems' versus ICQ side effect domains thought to be caused systemically e.g. 'Mood Problems' and 'Skin, Hair and Nails' domains).

#### Construct validity analyses

Using univariate analyses, we explored differences in ICQ scores between the four ICS dose groups (hypothesis 1). We also explored differences in other study variables, between the four ICS dose groups, in order to identify potential confounding variables (p-values ≤ 0.10), alongside those predicted by the research literature, for subsequent regression analysis (hypothesis 2). Non-normally distributed variables were tested using appropriate non-parametric tests, and for uniformity only the results of non-parametric test are reported as parametric test results were similar. Prevalence (scores > 0) of the 57 items on the ICQ were plotted onto graphs to show the dose-response pattern in reporting. Median (IQR) score and prevalence (scores > 0) were also tabulated for the total score and each domain of the ICQ. A multi-trait correlation matrix of the domains of the ICQ was calculated to determine the association among items in domains predicted to be caused via systemic or local routes of action (hypothesis 3).

Linear regression analysis was carried out to determine the extent to which the total ICQ score could be explained by daily ICS dose after adjusting for potential confounders. All variables were checked for normality prior to regression analysis. Given that ICQ scores were skewed with many zero values we took natural logs of the total ICQ score having initially added 0.5 to each score. This then served as the dependent variable in a multivariate model. In the regression model independent variables were ICS dose group, ACQ score and variables for which potential confounding was indicated. ICS dose group and ACQ score were first entered into the model followed by each single independent confounder in order to establish which variables showed independent associations with ICQ score. Finally a stepwise procedure was employed (following entry of ICS dose group and ACQ score) allowing simultaneous entry of the independent confounders. This analysis was subsequently repeated in those patients with well-controlled asthma as determined by the ACQ questionnaire (total ACQ score ≤ 0.75 [25]).

#### Reliability – reproducibility procedure and analysis

Test-retest reliability assesses the stability of a scale for producing reproducible results over time. 76 randomly selected construct validity patients, who were currently using an ICS inhaler, completed a second questionnaire after 7 days. The 7-day questionnaire included the ICQ scale, the 6-item ACQ, questions on medication change in the last 7 days, ICQ completion time (response options: less than 10 minutes, 10–15 minutes; 16–20 minutes; more than 20 minutes), missing side effects not included in the scale and perceived difficulty of the ICQ scale (response options: very difficult; difficult; not difficult; easy; very easy). Excluded patients reported a change in their ICS use or in any other medication from baseline measurement. Intraclass correlation coefficients (ICC) between baseline and follow-up ICQ scores were calculated to assess reproducibility.

#### Reliability – internal consistency procedure and analysis

Cronbach's alpha coefficient and item-total correlations were calculated to test the internal consistency of the ICQ.

### Study 2 – Responsiveness of the ICQ to changes in ICS use

Ethical approval for this study was obtained from Grampian Research Ethics Committee. Dutch ethical approval was sought but not required.

#### Patients

General Practitioners and Pulmonologists in North Netherlands and Aberdeen Scotland, invited by letter, agreed to recruit patients during any normal consultation which resulted in starting, increasing or decreasing their patients ICS dose by at least 400 μg. Patients were included in two countries to improve recruitment numbers and provide a representative sample of inhaler users. Physicians informed the researchers of the patients' old and new ICS prescriptions. Eligible patients were current inhaler users with physician diagnosed asthma, who gave informed consent, were aged ≥16 years, had not used oral steroids, received no change in their ICS prescription for 3 months prior to the study and received no further change during the 6-months of the study (subsequent to the physician-instigated dose change at study entry).

#### Responsiveness procedure

Participants completed a self-report questionnaire at baseline and follow up (2- and 6-months after ICS change), which measured: ICQ; 6-item ACQ (Forced expiratory volume in one second, question omitted); Asthma Quality of Life Questionnaire (AQLQ(S)[29]; daily ICS use and patient characteristics. We hypothesized that any change in ICS dose (higher or lower), would be associated with a reciprocal change in group side effect scores, for the total and domain scores of the ICQ.

#### Responsiveness analysis

We compared differences in ICQ, ACQ and AQLQ(S) scores at 2- and 6-months from baseline using the Wilcoxon test. The ICQ scores of patients who received a decrease in dose were reversed to analyze absolute change from baseline at 2-months and 6-months in the whole sample simultaneously. We produced a box plot of change in total ICQ scores (the difference (delta, Δ) between ICQ median total score at baseline and follow-up) by ICS dose change group (increased ICS versus decreased ICS). All statistical analyses in this article were performed with SPSS version 11.0 (SPSS Inc., Chicago, IL, U.S.A.).

## Results

### Study 1 – Domain construction, construct validity and reliability of the ICQ

#### Patients

Of 784 patients invited, 90% responded (n = 704). 318 were not eligible (no inhaler n = 239; oral or injected steroid n = 44; ≥20 pack years n = 25; questionnaire returned after closing date n = 8; questionnaire not filled in adequately n = 2). 131 did not wish to participate, leaving 255 eligible patients for analysis (see Table 1 for patient characteristics). The 131 non-participants were of similar age (median (IQR) 38 (26–49)) and gender (49% male) to the 255 participants included in the study (age 42 (33–50); 44% male).

**Table 1:** Patient characteristics and demographics by ICS daily dose group

Variable	Total Sample	Non ICS Inhaler	Low dose ICS ≤400 μg	Mid dose ICS 401–800 μg	High dose ICS > 800 μg	p-value
	n = 255	n = 27	n = 61	n = 62	n = 105	
Age (years)	42 (33–50)	33 (28–39)	40 (32–51)	47 (37–50)	42 (33–50)	0.005
% male	44	44	51	44	40	0.295*
						
**Smoking**						
% current or past smokers	44	37	44	47	43	0.775*
Pack years^a^	0 (0–3)	0 (0–2)	0 (0–4)	0 (0–4)	0 (0–2)	0.782
						
**Asthma severity**						
Asthma control questionnaire (ACQ) score (0–6)	0.8 (0.3–1.5)	1.2 (0.5–1.7)	0.5 (0.2–1.3)	0.7 (0.3–1.2)	0.8 (0.3–1.7)	0.075
No. emergency GP appointments for asthma in last year % none/% ≥1	80/20	85/15	87/13	84/16	73/27	0.034
						
**Educational level achieved**						
% Primary and lower vocational	34	26	31	34	37	
% Secondary/intermediate vocational	38	33	33	47	37	
% Higher vocational/university	28	41	36	19	26	0.070
						
**Use of asthma medication**						
No. of daily puffs SABA % none/% 1–2/% ≥3	69/24/7	22/59/19	79/20/1	84/10/6	68/25/7	0.049^¶^
Daily equivalent dose^† ^of LABA^c ^%0 μg/%1–200 μg	39/61	89/11	53/47	33/67	20/80	≤0.001
No. of courses of Prednisolone in last 3 years^c ^% none/%1–2/%≥3	57/27/16	74/19/7	64/27/9	58/27/15	49/29/22	0.002^¶^
% currently using nasal steroids	25	7	28	31	25	0.296*
% currently using dermal or ocular steroids	6	7	10	2	5	0.291*^¶^
No. of other concomitantly prescribed medications % none/%1–2/%≥3	37/42/21	52/30/18	39/48/13	34/48/18	34/39/27	0.048
						
**Perceived side effect in last 14-days:**						
ICQ total score	11 (4–22)	5 (0.8–11)	7 (3–14)	10 (3–21)	15 (9–30)	≤0.001
						
**Personality:**						
Negative affectivity score (PANAS)	17 (14–22)	14 (13–20)	15 (13–21)	17 (14–21)	17 (15–24)	0.09
Neuroticism score (EPQ-RSS) score (0–12)^d^	4 (2–7)	4 (1–7)	3 (1–6)	4 (2–6)	4 (1–7)	0.727*
						
**Inhaler behavior**						
% rinsing mouth after inhalation^e^			75	82	77	0.928*
% using spacer device			2	7	15	0.003
% use of ICS 7 days per week (adherent)^b^			82	90	99	≤0.001

*Variable entered into regression due to potential confounding indicated by research literature

Variable not entered into regression as ^a^variable 'current or past smokers' entered into model ^b^variable used to calculate μg daily ICS dose

Missing data: ^c^n = 7, ^d^n = 2, ^e^n = 1

^†^Salmeterol equivalent dose: 4 μg salmeterol = 1 μg formoterol (only 4% of total sample used >100 μg daily)

All variables % or median (IQR), tested with Chi-square (p-value linear trend) or Kruskal-Wallis except ^¶ ^Fishers Exact Test (p-value linear trend)

#### ICQ domain construction

Eight factors were identified using eigenvalues and the Cattell Scree plot. Four items were constructed into 2 additional domains using inter-item correlations, leaving the remaining 5 items to be formed into single item domains. The final 14-day ICQ questionnaire therefore consists of 15 domains (see Table 2).

#### ICQ scoring

The ICQ was scored on an item level from 0 (not at all) to 6 (a very great deal) other response options being: 1 (a very little), 2 (a little), 3 (a moderate amount), 4 (quite a lot), 5 (a great deal). In order that domain scores with differing numbers of items could be compared, the 15 domain scores were transformed into a score out of 100 ((Raw domain score/(6 * no. items in domain)) *100). The total score of the ICQ (0–100) was the average of the 15 domain scores (sum the scores of 15 domains/15). The highest score represents the greatest side effect.

### Endorsement and scaling

Endorsement frequency (i.e. the percentage of patients scoring ≥1) for the 57 items in the ICQ scale ranged from 13% to 64%. Endorsement frequency was less than the recommended 20% [18] for 4 items on the scale: 'oral thrush' (13%), 'loss of ability to taste' (19%), 'a loss of appetite' (15%) and 'swollen face or fluid around the face' (15%). Notwithstanding this, we left the 4 items in, since we recommend a less stringent approach to item endorsement for a side effect scale where even low percentages of reporting on the group level might be of significant clinical importance. With respect to scaling (that is, the use of each of the seven response options on the ICQ Likert scale e.g. "not at all" to "a very great deal"), no single response option for the ICQ scale was responded to by ≥95% of participants.

### Construct validity

A number of variables showed a linear relationship with ICS dose group (Table 1); which may have confounded scoring on the ICQ. These variables were age, asthma control, emergency GP appointments for asthma in last year, educational level, number of daily puffs of SABA, daily dose of LABA, courses of Prednisolone in the last 3 years, number of concomitantly prescribed medications, negative affectivity score, and use of a spacer device. Variables which showed no linear trend, but were potential confounders indicated by the research literature, were gender, smoking status, use of nasal steroids, neuroticism score, and mouth rinsing after inhalation. Age when asthma diagnosed, number of comorbidities, and rhinitis diagnosis, were also assessed (data not shown) but were not significantly different between groups.

#### Hypothesis 1

**Figure 1:**
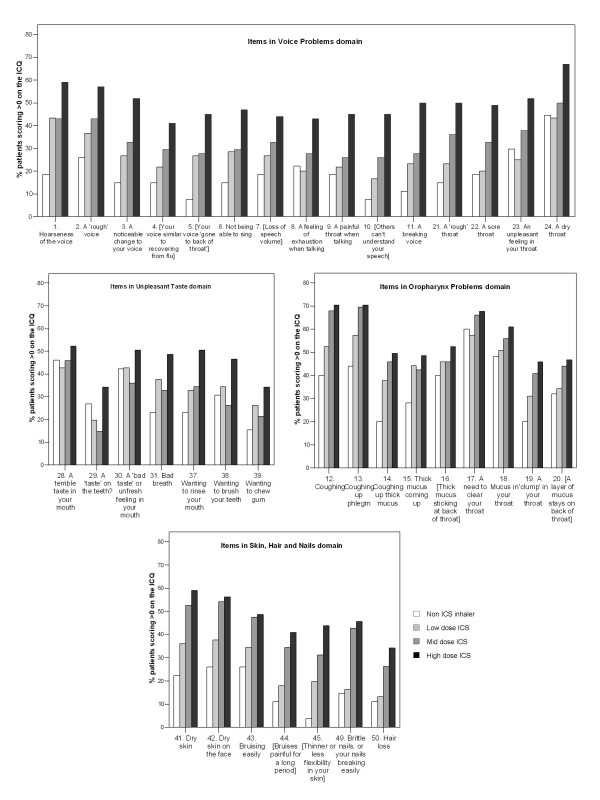
**Prevalence of items in the 'Voice Problems', 'Unpleasant Taste' 'Oropharynx Problems' and 'Skin, Hair and Nails' domains of the ICQ**. Square brackets indicate truncation of text – full item description in table 2

**Figure 2:**
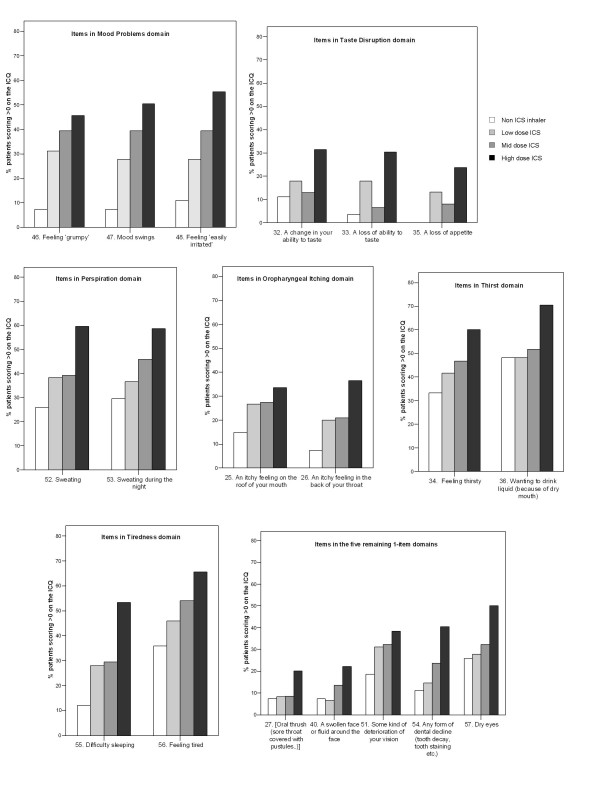
**Prevalence of items in the 'Mood Problems', 'Taste Disruption', 'Perspiration', 'Oropharyngeal Itching', 'Thirst', 'Tiredness' and five 1-item domains of the ICQ**. Square brackets indicate truncation of text – full item description in table 2

A dose-response was observed for all 57 items on the ICQ, when comparing high- versus low-dose ICS groups (Figures 1 to 2). However the mid-dose group showed a lower prevalence than the low-dose ICS group in the 'Unpleasant Taste' domain (items 29, 30, 31, 38, 39), 'Oropharynx Problems' domain (item 15) and the 'Taste Disruption' domain (items 32, 33, 35). ICQ total and domain scores showed hierarchical differences between dose groups (with the highest scoring in the high dose ICS group) that were statistically significant for 14 of the 15 domains (Table 3). Only the 'Vision Deterioration' domain did not reach statistical significance in this sample, although there was a suggestion of an effect (p = 0.066).

**Table 3:** Median score and prevalence by ICS dose group for ICQ total and 15 domains

ICQ Domain	Non ICS Inhaler n = 27	% >0	Low dose ICS n = 61	% >0	Mid dose ICS n = 62	% >0	High dose ICS n = 105	% >0	p-value†
Total Score	5 (1–11)	[89]	7 (3–14)	[92]	10 (3–21)	[92]	15 (9–30)	[97]	<0.001
Voice Problems	2 (0–8)	[56]	3 (0–13)	[70]	6 (0–22)	[63]	13 (3–34)	[79]	<0.001
Oropharynx Problems	11 (0–19)	[67]	9 (1–28)	[75]	18 (3–41)	[81]	20 (6–44)	[86]	0.003
Unpleasant Taste	7 (0–24)	[67]	5 (0–20)	[61]	5 (0–19)	[68]	10 (2–29)	[77]	0.012
Skin, Hair and Nails	0 (0–14)	[41]	5 (0–19)	[57]	14 (0–24)	[69]	19 (5–37)	[81]	<0.001
Mood Problems	0 (0–0)	[11]	0 (0–19)	[33]	0 (0–33)	[46]	11 (0–39)	[59]	<0.001
Taste Disruption	0 (0–0)	[11]	0 (0–0)	[23]	0 (0–0)	[16]	0 (0–14)	[39]	0.001
Perspiration	0 (0–25)	[30]	0 (0–33)	[45]	0 (0–38)	[46]	17 (0–42)	[64]	0.002
Oropharyngeal Itching	0 (0–0)	[15]	0 (0–8)	[27]	0 (0–8)	[32]	0 (0–25)	[41]	0.002
Thirst	0 (0–25)	[48]	8 (0–25)	[55]	8 (0–35)	[58]	25 (0–50)	[72]	<0.001
Tiredness	0 (0–13)	[36]	8 (0–25)	[51]	8 (0–33)	[57]	25 (0–42)	[74]	<0.001
Oral Candidiasis	0 (0–0)	[7]	0 (0–0)	[8]	0 (0–0)	[8]	0 (0–0)	[20]	0.014
Facial Oedema	0 (0–0)	[7]	0 (0–0)	[7]	0 (0–0)	[13]	0 (0–0)	[22]	0.005
Vision Deterioration	0 (0–0)	[19]	0 (0–17)	[31]	0 (0–17)	[33]	0 (0–33)	[38]	0.066
Dental Deterioration	0 (0–0)	[11]	0 (0–0)	[15]	0 (0–13)	[25]	0 (0–33)	[40]	<0.001
Eye Dryness	0 (0–17)	[26]	0 (0–17)	[28]	0 (0–17)	[31]	0 (0–50)	[50]	0.001

† Jonckheere-Terpstra Test

ICQ score median (IQR)

ICQ domain and total scores: 0 to 100

[% patients scoring % >0 within ICQ domain]

#### Hypothesis 2

ICS dose group remained an independent predictor of ICQ score (Table 4) in the stepwise linear regression analyses, after adjusting for potential confounders: the explained variance being 35.8%. Subsequent multiple regression analysis in 124 patients with well-controlled asthma (non ICS inhaler n = 9; low dose ICS n = 34; mid dose n = 34; high dose n = 47), showed that only ICS dosage group (p < 0.001) and neuroticism score (p = 0.007) were independent predictors of ICQ score (data not shown).

**Table 4:** Stepwise regression model of factors influencing ICQ total score

Model	B-coefficient	Standard error	p-value
ACQ score	0.43	0.07	<0.001
Gender*	-0.42	0.15	0.007
ICS dose group	0.37	0.07	<0.001
Neuroticism score	0.09	0.03	0.001
Number of concomitant medications used	0.21	0.10	0.038

*Female gender associated with higher side effect scoring

Dependent variable: logged ICQ total score

#### Hypothesis 3

The multi-trait correlation matrix (Table 5) showed that associations between items in side effect domains with a potentially homogenous route of action were greater (r = 0.52; r = 0.39) than those between items in domains with a potentially heterogeneous route (all ≤0.32).

**Table 5:** A multi-trait correlation matrix to test convergence between the side effect domains of the ICQ

	1	2	3	4
1. Voice Problems	(0.65)			
2. Oropharynx Problems	**0.52**	(0.69)		
3. Skin, Hair and Nails	*0.27*	*0.28*	(0.50)	
4. Mood	*0.32*	*0.32*	**0.39**	(0.87)

R-values in bold show domains with a potentially homogenous route of action which should be higher than correlation coefficients in italics which show the heterogeneous domains tested. Remaining coefficients in brackets show the inter-item correlation for each domain

#### Reliability – internal consistency

For the 57 items in the scale, all item-total correlations were greater than r = 0.20 as recommended by Kline 1986 (in [18]). Cronbach's Alpha was 0.98, which was well above the minimum (α = 0.70) recommended by Nunnally (1978) (in [18]) for internal consistency.

#### Reliability – reproducibility and patient acceptability

73 participants returned their second questionnaire. 68 patients had made no changes to their use of ICS or other medication since baseline measurement. We had planned to exclude patients if their ACQ score changed by more than 0.5 from baseline (the scales minimal clinically important difference [25]), but ICC for the whole sample (n = 68) compared to those without a change in ACQ score (n = 48) showed no detrimental impact upon the reliability of the ICQ, so results for all 68 patients are shown. ICC for 14 of the 15 domains were greater than >0.5 as recommended for reproducibility coefficients [18] (Table 6). Only the domain 'Facial Oedema' was slightly less stable over 7 days (r = 0.41). The majority of patients (88%) completed the questionnaire in less than 15 minutes, and 91% reported that the questionnaire was simple to fill in. Four additional side effects were suggested, each by one patient: pigment spots, fragile bones, itch in nose, and problems with kidney and liver.

**Table 6:** ICC for 7 day test-retest of the ICQ

ICQ Domain	No. of items in domain	ICC	% patients scoring zero at baseline and follow-up
Total Score		0.91	6
Voice Problems	15	0.89	24
Oropharynx Problems	9	0.87	12
Unpleasant Taste	7	0.91	24
Skin, Hair and Nails	7	0.81	15
Mood Problems	3	0.73	41
Taste Disruption	3	0.80	56
Perspiration	2	0.75	35
Oropharyngeal Itching	2	0.83	61
Thirst	2	0.84	30
Tiredness	2	0.79	30
Oral Candidiasis	1	0.82	79
Facial Oedema	1	0.41	77
Vision Deterioration	1	0.80	55
Dental Deterioration	1	0.69	61
Eye Dryness	1	0.81	54

### Study 2 – Responsiveness of the ICQ to changes in ICS use

#### Patients

82 General Practitioners volunteered to recruit patients (67 North Netherlands (N), 15 Aberdeen, Scotland (S)), but, after 1 year, practices produced low patient numbers in both countries, predominantly due to low frequency of ICS prescription change in general practice. We decided to rationalize participating general practices to the 33 most motivated (26 N, 7 S) and widened our recruitment net by inviting an additional 6 secondary care physicians to recruit patients in The Netherlands.

94 eligible patients were recruited at baseline (90 N; 4 S), yet 16 deviated from prescribed ICS dose, 8 used oral steroid, 5 dropped out (3 too busy, 1 illness, 1 stopped ICS due to voice side effects), 3 were lost to follow up, and 1 could not complete the questionnaire. Thus we were able to analyze data of 61 patients at 2-months (58 N; 3 S). We could analyze 39 patients at 6 months, since a further 19 deviated from ICS dose change, 1 dropped out (illness) and 2 used oral steroids (36 N; 3 S). Of the 39 participants at 6-months, 21 patients (median age 50 (IQR 41, 65), 57% female, 91% mouth rinsing after inhalation, 57% using budesonide) had received a median ICS dose increase of 800 μg (IQR 400, 800), and 18 patients (median age 57 (IQR 51, 63), 67% female, 100% mouth rinsing after inhalation, 56% using budesonide) had received a median ICS dose decrease of 450 μg (IQR -650, -400).

#### Responsiveness

**Figure 3:**
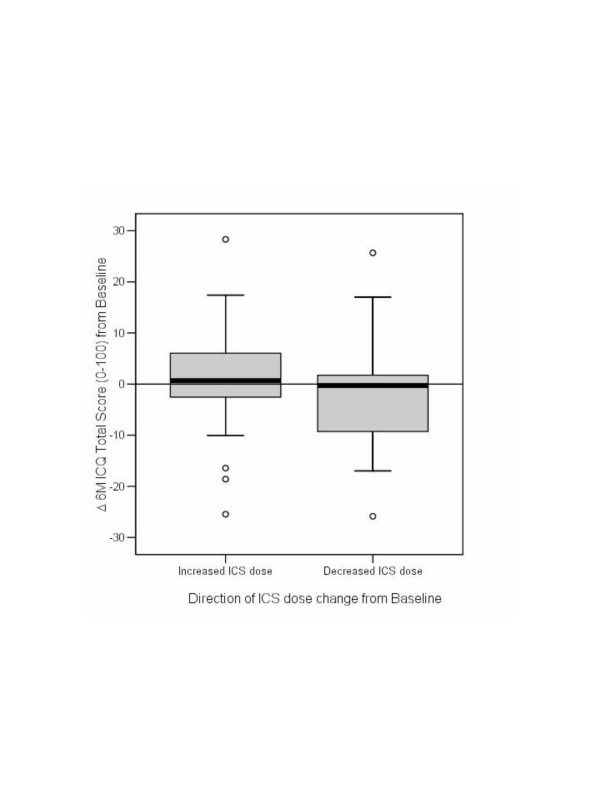
Change in total ICQ score from baseline for Increased ICS and Decreased ICS dose groups.

At 2-months from baseline, differences were seen in the 'Voice Problems' domain (p = 0.023). However, at 6 months from baseline the statistically significant difference in the domain 'Voice Problems' (p = 0.026) remained and the domains 'Skin, Hair and Nails' (p = 0.064) and 'Facial Oedema' (p = 0.056) showed some evidence of an effect (Table 7). Non-significant trends were also observed for 'Perspiration' (p = 0.109), 'Unpleasant Taste' (p = 0.155) and 'Dental Deterioration' (p = 0.185). Scores for other domains showed less clear scoring trends during the study. Total scores varied widely within groups, but a trend was observed in Δ ICQ total score (the difference (delta, Δ) between ICQ median total score at baseline and 6-months measurements) relative to dose change direction at 6-months (Figure 3), but not at 2-months (data not shown).

**Table 7:** Change in ICQ score at 6-months for increased ICS and decreased ICS dose groups

ICQ score	Increased ICS Dose (n = 21)		Decreased ICS Dose (n = 18)		
	BL	6M	BL	6M	p-value*
Total Score	8 (3–26)	16 (7–24)	16 (8–28)	11 (5–31)	0.460
Voice Problems	8 (2–25)^†^	13 (4–37)	14 (6–36)	13 (2–22)^†^	**0.026**
Oropharynx Problems	24 (7–39)	30 (11–42)	29 (12–38)	13 (6–33)	0.330
Unpleasant Taste	5 (0–25)	17 (4–29)	8 (0–20)	5 (0–24)	0.155
Skin, Hair and Nails	7 (0–27)	14 (1–33)	23 (13–31)	11 (4–36)	0.064
Mood Problems	6 (0–31)	0 (0–22)	3 (0–33)	0 (0–19)	0.455
Taste Disruption	0 (0–28)	6 (0–17)	0 (0–18)	0 (0–17)	0.379
Perspiration	17 (0–42)	17 (0–42)	17 (0–38)^†^	17 (0–27)	0.109
Oropharyngeal Itching	0 (0–21)	0 (0–17)	0 (0–10)	0 (0–19)	0.533
Thirst	17 (0–50)^†^	25 (0–42)	17 (0–29)	13 (0–42)	0.593
Tiredness	17 (0–46)	25 (0–33)	33 (0–52)	29 (0–52)	0.465
Oral Candidiasis	0 (0–17)	0 (0–17)	0 (0–33)	0 (0–17)	0.403
Facial Oedema	0 (0–0)	0 (0–17)	0 (0–17)	0 (0–0)	0.056
Vision Deterioration	0 (0–33)	0 (0–33)	0 (0–33)	0 (0–17)	0.756
Dental Deterioration	0 (0–0)	0 (0–17)^†^	0 (0–17)	0 (0–0)^†^	0.185
Eye Dryness	0 (0–17)	0 (0–17)	33 (0–50)	17 (0–50)	0.915

* Wilcoxon test: p-values for change in absolute values of ICQ scores for all patients at 6M from BL

† data missing in domain for 1 patient

All data median (IQR)

At 6-months, asthma control and asthma-related quality of life improved significantly from baseline in the group whose ICS increased, but not for the group whose ICS dose decreased (Table 8). Change at 2-months was similar (data not shown).

**Table 8:** Change in asthma control and asthma quality of life scores at 6-months for increased ICS and decreased ICS dose groups

Scale	Increased ICS Dose (n = 21)		Decreased ICS Dose (n = 18)	
	BL	6M	BL	6M
ACQ Total Score	1.5 (0.8, 2.3)	1.2 (0.7, 1.8)**	0.7 (0.3, 1.3)	0.8 (0.2, 1.4)
AQLQ(S) Total Score	5.1 (4.5, 5.9)	5.7 (5.1, 6.3)*^†^	6.0 (5.3, 6.6)	6.3 (5.5, 6.8)

All data median (IQR); ACQ (0–6) scored: 0 = best control, 6 = worst control; AQLQ(S) (7–1) scored: 7 = best, 1 = worst

Change from baseline tested with Wilcoxon *p = 0.001 ** p = 0.039

†Clinically important change (≥0.5 on scale)

## Discussion

Medication-related side effects are of considerable concern to patients and may result in their non-compliance to prescribed regimens. Despite this, at the present time no method exists for the systematic measurement of patients' perceptions of inhaled corticosteroid side effects. Drug-specific self-report questionnaires could provide a simple method for communicating patients' side effect experiences to their physician in everyday practice, during clinical trials, and in new studies paramount for establishing the impact of side effect on ICS adherence. In this paper, using a combination of well-established methods, we first constructed domains of the Inhaled Corticosteroid Questionnaire (ICQ) and then tested the validity, reliability and initial responsiveness of the scale.

### Study 1 – Construct validity and reliability

Our test of construct validity was based on a series of a priori set hypothesis which were all confirmed. A dose-response was observed for reporting of all 57 items on the scale and ICQ total and domain scores were statistically significantly different between ICS dose groups. Of fundamental importance, scoring on the ICQ was predicted by current daily ICS dose even after controlling for potential confounding variables in regression analysis. Specifically, this meant that after accounting for female gender, asthma control, neuroticism and use of other medications, which also predicted higher scores, higher ICS dose still independently and significantly predicted higher total ICQ scores. Furthermore, associations among ICQ domains supported the structural functioning of the scale. Side effect domains of the ICQ thought to be caused by the same pathway (action of glucocorticoids via oropharyngeal deposition) such as 'Voice Problems' and 'Oropharynx Problems' were more highly correlated than those thought to be caused via different routes of action, such as 'Voice Problems' and 'Mood'(action of glucocorticoids via absorption into the systemic circulation). These combined results show that the ICQ has good construct validity.

A few construct validity results require further discussion. The 'Vision Deterioration' domain of the ICQ showed evidence of a dose effect, with prevalence in the high ICS dose group (38%) double that of non ICS users (19%), but overall differences between groups for this domain did not reach statistical significance (p = 0.066). The clinical importance of vision deterioration as a marker for potential ICS-related glaucoma or cataract and the differences observed between ICS users and non-users support its retention in the scale.

Higher neuroticism score consistently predicted higher ICQ scoring in this sample providing further evidence for the validity of the scale. Neuroticism is a personality factor related to the propensity of experiencing emotional distress, poorer stress coping, higher ratings of poor health [30] and increased reporting of symptoms [31,32]. It therefore follows that persons high in neuroticism are also likely to self-report greater side effects of medication, and this is of considerable interest for future research.

It is possible that part of the dose-response observed in ICQ scoring may reflect disease symptoms rather than medication-related side effects. Because asthma patients with worse symptoms generally have the worst asthma control and are usually prescribed the highest ICS doses, we presumed that asthma control would be related to ICQ scoring. For this reason, we wished to test if the relationship between ICS dose and ICQ still existed when asthma control was no longer a predictor of ICQ scoring. Regression analysis in a subgroup of 124 patients with homogenous asthma (ACQ score ≤ 0.75) showed that ICS dose continued to explain the largest part of variation in ICQ scoring after adjusting for potential confounders. Neuroticism also remained an important predictor. Together this is suggestive evidence that the ICQ scale measures side effects independently of disease control.

Our 7-day test re-test reliability data supports that the ICQ is a reliable scale with good reproducibility over time. However, the frequency of zero scoring at baseline and 7-day follow-up may have augmented some reproducibility coefficients. It is recommended that reproducibility coefficients should be greater than 0.5 [18], and only one of the 15 domains, i.e., 'Facial Oedema', was less stable over 7 days (r = 0.41). Without treatment, side effects can spontaneously resolve themselves during repeated measurement. Facial oedema may be particularly vulnerable to change over the relatively long re-test period of 7 days, due to extraneous factors (such as allergy). Facial oedema is however a recognized corticosteroid side effect and since its validity was acceptable, the item is retained to protect the content validity of the scale.

The ICQ showed excellent internal consistency in this study (α = 0.98). This reliability coefficient is relatively high, although not exceptional, as quality of life questionnaires often produce alphas of well above 0.9 (Airways Questionnaire 20 [33]; St. George's Respiratory Questionnaire, Chronic Respiratory Disease Questionnaire [34]). Side effect scales require good content validity (including the range of potential side effects experienced), which can simultaneously result in a lengthy scale and a relatively high level of zero scoring which together inflate alpha scores. The ICQ scale also shows appropriate item endorsement (that is for every item on the scale ≥13% of patients scored ≥1), and scale endorsement (that is, less than 95% of patients used the same response option on the ICQ's Likert scale e.g. "not at all" or "a very great deal"). The ICQ was also acceptable to patients, completed without difficulty (91% of patients) in 15 minutes or less (88% of patients), demonstrating the viability of the scale for use in clinical practice or research.

Results should be considered in the context of our research. All data in the study was necessarily self-reported and the possibility of bias exists. We considered, but finally rejected, the inclusion of an objective measure of disease severity (e.g. lung function) in this study, since the association between lung function and patients' self-reported disease symptoms is rather tenuous [35-37], and unlikely to provide information in addition to the Asthma Control Questionnaire. Since our study population had used ICS for a relatively long period (median (IQR): 11 (7–20) years), side effect reporting in this sample could have become diminished due to habituation to side effect over time, or conversely, augmented by an increased awareness of side effect or the cumulative effect of long-term ICS use. Our scale must necessarily measure the side effect perceptions of patients, and the favorable validity and reliability results we achieved in this sample, strengthen our confidence in the scale. Our initial response rate was very good (90%), which may reflect the particularly compliant characteristics of our cohort explaining, in part, their continued long-term use of ICS regardless of the side effects they reported. For this reason further validation work in different samples, in other diseases for which ICS are prescribed, such as Chronic Obstructive Pulmonary Disease, and in different countries would be useful to add to our results.

### Study 2 – Responsiveness of the ICQ to changes in ICS use

In our responsiveness study we compared ICQ scores at baseline, to those at 2- and 6-months after a change in ICS dose. The questionnaire showed potential evaluative properties, especially for the domains 'Voice Problems' at 2- and 6-months and 'Skin, Hair and Nails' and 'Facial Oedema' at 6-months. The difference in temporal responsiveness between these local and systemically caused side effects seems consistent with differences in the emergence of side effects on starting ICS: dysphonia can occur within a few days, whereas skin atrophy can occur six weeks after starting treatment [38,39].

Our results should be considered in the context and limitation of the research design. Although we did not have the opportunity to test the ICQ in a randomized controlled design, which would have allowed us to rule out regression to the mean or reporting bias, the fact that trends in ICQ score change were largely reciprocal to dose modulation and that change was stronger at 6- than at 2-months, supports its potential evaluative properties. A longer follow-up period may have been needed to allow more time for side effects to respond to dose change, and our observational design resulted in a high sample attrition which occurred largely due to deviation from prescribed ICS dose changes. This may explain our inability to detect a statistically significant change in this study. However this does not rule out potentially clinically important change, since at 6-months the total ICQ score showed a two-fold increase from baseline in the group whose ICS dose had been increased. Moreover, it is unlikely that modulation in asthma symptoms were responsible for changes in side effect scores in this study, since change in asthma control was not reciprocal to ICQ scoring, in fact asthma control improved in the ICS increasers group whose side effect worsened.

Temporal variation in the appearance of different side effects [40] and differential responses to a change in dose of medication (in number, duration or severity of side effects) [5] in different individuals, can hamper responsiveness testing. One must take care not to over-interpret results before a large number of tests have been undertaken. Additional assessment, ideally in a randomized controlled trial, must be carried out to confirm the responsiveness of the ICQ.

## Conclusion

We have developed the Inhaled Corticosteroid Questionnaire; a useful new tool for measuring patients' perceptions of ICS associated side effects. The ICQ shows good reliability and validity in asthma patients and has good discriminative properties. It also has potential evaluative properties that require further testing. Used for systematic measurement, this simple questionnaire may provide a greater understanding of the side effect perceptions of patients in clinical practice and clinical trials, and will yield useful new perspectives on the relationship between perceived side effect of ICS and adherence.

## Abbreviations

ACQ = Asthma Control Questionnaire; AQLQ(S) Asthma Quality of Life Questionnaire (Standardized); BDP = Beclomethasone dipropionate; EPQ-RSS = Eysenck Personality Questionnaire Revised Short Scale; ICC = Intraclass correlation coefficients; ICQ = Inhaled Corticosteroid Questionnaire; ICS = Inhaled Corticosteroids; IQR = Inter-Quartile Range; LABA = Long-acting β_2_-agonist; PANAS = Positive and Negative Affect Schedule; SABA = Short-acting β_2_-agonist

## Competing interests

The author(s) declare that they have no competing interests.

## Authors' contributions

JMF participated in the design of the study, collected the data, performed the statistical analysis and drafted the manuscript. EVS AJL and RS participated in the study design, supervised statistical analysis and assisted in the interpretation of statistical results. AD participated in the study design and data collection. DSP supervised and designed all cohorts used in study 1, assisted in collecting phenotype data of study 1 cohorts, conceived of the study, participated in the study design and assisted in the drafting of the manuscript. TVM conceived of the study, participated in the study design and assisted in the drafting of the manuscript. All authors read, commented on and approved the final manuscript.
